# Stability of Genomic Selection prediction models across ages and environments

**DOI:** 10.1186/1753-6561-5-S7-O14

**Published:** 2011-09-13

**Authors:** Marcio FR Resende, Patricio R Muñoz Del Valle, Juan J Acosta, Marcos DV Resende, Dario Grattapaglia, Matias Kirst

**Affiliations:** 1 Genetics and Genomics Graduate Program, University of Florida, Gainesville, FL, USA; 2 Plant Molecular and Cellular Biology Graduate Program, Gainesville, FL, USA; 3 School of Forest Resources and Conservation, University of Florida, Gainesvile, FL, USA; 4 EMBRAPA Forestry, Colombo, Parana, Brazil; 5 EMBRAPA Genetic Resources and Biotechnology, Brazilia, DF, Brazil

## Background

A tree breeding program is characterized by long generation intervals which, over time, result in a much smaller number of breeding cycles when compared to annual crops. Moreover, most economically important traits in a tree-breeding program are quantitatively inherited, display low heritability and are expressed late in the life cycle. Genomic Selection (GS) is expected to be particularly valuable for tree species, leading to shorter generation intervals and improved genetic gain over time.

The main factors that affect the accuracy of GS prediction models are the level of linkage disequilibrium (LD) in the training population, the training population size, the heritability of the trait and the number of QTL regulating its variation. However, it is yet largely unknown how stable prediction models are across environments and different ages. This knowledge is critical for tree breeders that wish to use genomic selection in their genetic improvement program.

Here, we report the first assessment of the utility of genomic selection in a conifer species. We developed prediction models for growth traits measured at multiple sites, to evaluate the impact of genotype by environment interactions in their accuracy. Training populations were also measured over multiple ages and models were developed to assess their value in predicting breeding values later in the lifecycle.

## Material and methods

Here we analyzed a population of 790 to 840 individuals of loblolly pine, clonally replicated in four sites in the southeastern US: Palatka and Nassau (Florida, USA), Cuthbert and B.F. Grant (Georgia, USA). The population is derived from 61 full-sib families, established by crossing 32 parents in a circular mating design. The traits analyzed in this study were diameter at breast height (DBH) measured when trees were three, four and six years old; and total height (HT) measured when trees were one, two, three, four and six years old. All the individuals were genotyped with a total of 3,938 SNPs [1]. Single marker regression association analyses were initially performed treating the markers as fixed effects. The markers that were selected in this association analysis had their effects estimated adjusting all the allelic effects simultaneously using a genomic BLUP procedure [2]. These analyses were performed across all sites, traits and ages and the estimated effects of the markers were validated using a 10-fold cross validation approach. The selection gain of genomic selection was compared to classical phenotypic selection considering a reduced breeding cycle due to early selection.

## Results and discussion

The accuracies of the prediction models for GS developed using phenotypic data measured in each site at year 6 ranged from 0.65–0.75 for DBH, and 0.64–0.77 for HT. To evaluate the performance of GS relative to traditional breeding methods, we estimated the accuracies of BLUP-based selection [3] and used it as a benchmark for the comparison of the accuracies obtained by GS. The increase in efficiency per unit of time in the selection response of GS was 53–95% higher for DBH, and 58–118% higher for HT, assuming a conservative reduction of 50% in the length of the breeding cycle.

To evaluate if models generated at early ages would predict well the phenotype at mid-rotation, we assessed the accuracy of models developed for HT based on data collected at ages 1 to 4, but validated with measurements from the same populations at age 6. Accelerating model estimation is beneficial because the sooner models that accurately predict phenotypes at rotation age can be developed, the faster genomic selection can be adopted. However, the models developed for HT early in the rotation (age 1 to 3) had limited accuracy in predicting phenotypes at age 6.

Next, we tested the suitability of models estimated in each individual site, in predicting phenotypes across different sites. The accuracies reduced up to 86% (Table 1) and the decrease parallels the increase in geographic distance between the site for which models were estimated, and the site where they were validated. Therefore, environment × genotype interactions appear to severely affect the transferability of models across breeding zones.

**Figure 1:**
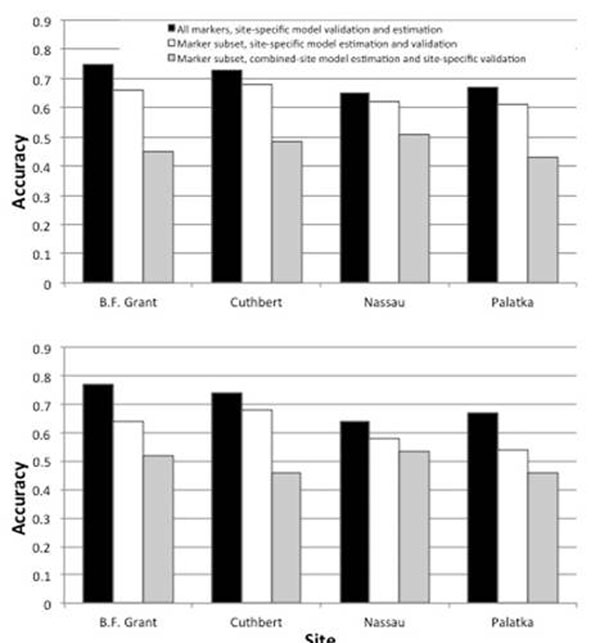
Accuracy of genomic selection models estimated using the complete (black bar) or subset of markers significant across all sites (white and grey bars) for DBH (upper panel) and HY (lower panel). Prediction models were estimated and validated in each individual site (black and white bars), or estimated based on a combined-site model and validated in idiviudal sites (grey bar).

Out of the total number of markers, 177 and 83 were significantly associated with traits across all sites for DBH and HT, respectively. Those were selected for model estimation since they may reflect marker-trait associations that are independent, or at least less affected, by genotype × environment interactions. Initially, models were re-estimated based on data from each site, using only the subset of markers, and re-validated in the same site. Accuracies were only slightly lower (3–9% for DBH, 6–13% for HT) than those obtained when models included the full set of significant makers (Figure 2). Therefore, a significant component of the genetic variance appears to be captured by relatively few markers (~100). Next, prediction models were estimated based on a combine-site model and validated individually in each of the four sites (Figure 1). While accuracies remained relatively high across sites (0.43–0.51 for DBH, 0.46–0.54 for HT), there is a decrease of up to 30% relative to the accuracy obtained when the full set of markers and site-specific specific data was used for model estimation (Figure 1). Therefore, while the same reduced set of markers is relevant for explaining the genetic variation in all sites, the magnitude of their effect changes significantly from site to site.

In conclusion, the results in efficiency demonstrated that incorporating genomic selection would dramatically increase the genetic gains per unit of time of a conifer’s breeding program. Moreover, even at relatively low marker density, the accuracy of prediction models could significantly impact the genetic gain efficiency. However, the use of a prediction model should be constrained within a breeding zone once genotype x environment can affect the prediction and reduce the accuracy of those models.
